# Optogenetic control of gene expression in the cyanobacterium *Synechococcus* sp. PCC 7002

**DOI:** 10.3389/fbioe.2024.1529022

**Published:** 2025-01-17

**Authors:** Liam Forbes, Maria Papanatsiou, Anna Palombo, John M. Christie, Anna Amtmann

**Affiliations:** School of Molecular Biosciences, College of Medical, Veterinary and Life Sciences, University of Glasgow, Glasgow, United Kingdom

**Keywords:** cyanobacteria, optogenetics, biotechnology, CcaS/CcaR, YF1/FixJ, gene expression, promoter, synthetic biology

## Abstract

Photosynthetic cyanobacteria can be utilised in biotechnology as environmentally sustainable cell factories to convert CO_2_ into a diverse range of biochemicals. However, a lack of molecular tools available for precise and dynamic control of gene expression hinders metabolic engineering and contributes to low product titres. Optogenetic tools enable light-regulated control of gene expression with high tunability and reversibility. To date, their application in cyanobacteria is limited and transferability between species remains unclear. In this study, we expressed the blue light-repressible YF1/FixJ and the green/red light-responsive CcaS/CcaR systems in *Synechococcus* sp. PCC 7002 and characterised their performance using GFP fluorescence assays and qRT-PCR. The YF1/FixJ system of non-cyanobacterial origin showed poor performance with a maximum dynamic range of 1.5-fold despite several steps to improve this. By contrast, the CcaS/CcaR system originating from the cyanobacterium *Synechocystis* sp. PCC 6803 responded well to light wavelengths and intensities, with a 6-fold increased protein fluorescence output observed after 30 min of green light. Monitoring GFP transcript levels allowed us to quantify the kinetics of transcriptional activation and deactivation and to test the effect of both multiple green/red and light/dark cycles on system performance. Finally, we increased CcaS/CcaR system activity under green light through targeted genetic modifications to the *pCpcG2* output promoter. This study provides a detailed characterisation of the behaviour of the CcaS/CcaR system in *Synechococcus* sp. PCC 7002, as well as underlining the complexity of transferring optogenetic tools across species.

## 1 Introduction

Cyanobacteria are photosynthetic bacteria that can be exploited as an environmentally sustainable cell chassis to produce biochemicals. More commonly used heterotrophic cell chassis such as *Escherichia coli* or *Saccharomyces cerevisiae* rely on sugar feedstocks as a carbon source for growth, which are often synthetically produced or derived from food crops (Muñoz et al., 2014). In contrast, cyanobacteria use light and CO_2_ as feedstock to synthesise a range of products with commercial applications in low-carbon or carbon-negative bioprocesses. These products include native compounds such as pigments and exopolysaccharides (Roy and Pabbi, 2022; Madsen et al., 2023), as well as non-native products through genetic engineering such as biofuels (Liang et al., 2018).

Despite great potential, the application of cyanobacteria for industrial biochemical production is not widespread. Often, cyanobacteria are unable to compete economically with the chemical synthesis of compounds or with production via heterotrophic cell chassis due to slow growth rates and low product titres (Gale et al., 2019). One route to improving product titres is to regulate the expression of individual enzymes during metabolic engineering. While chemically inducible promoters are often used to control the timing of enzyme expression, their use causes irreversible activation of gene expression, preventing more dynamic metabolic pathway control strategies from being applied.

Optogenetic tools enable tuneable and reversible control of gene expression using different wavelengths of light, which is inexpensive and non-invasive (Ohlendorf and Möglich, 2022). As light can easily be applied to or removed from cells in varying qualities and doses it allows flexible transcriptional regulation of target genes and opens new opportunities for control over metabolic pathways. This contrasts with the irreversible activation of transcription imparted by chemically inducible promoters, whilst also removing the need for expensive chemical inducer molecules such as isopropyl β-D-1-thiogalactopyranoside (IPTG). Optogenetics is rooted in the function of photoreceptor proteins which undergo conformational changes when absorbing specific light wavelengths. The conformational change triggers the activation or deactivation of an effector domain. Effector domains are often DNA-binding domains in the case of one-component systems, or histidine kinase domains which control the activity of a transcriptional regulator via phosphorylation in the case of two-component systems (Lindner and Diepold, 2022).

While optogenetic tools have been used in many heterotrophic cell chassis including *S. cerevisiae*, *E. coli*, *Bacillus subtilis* and *Pichia pastoris*, their application in cyanobacteria to date is limited (Sorokina et al., 2009; Tabor et al., 2011; Castillo-Hair et al., 2019; Wang et al., 2022). The ability to reversibly modulate enzyme expression in cyanobacteria via optogenetics could lead to increased product titres *versus* chemically inducible systems, as has been demonstrated in *S. cerevisiae* (Zhao et al., 2021). Furthermore, the transferability of optogenetic tools across species and strains remains unexplored. One of the few examples of optogenetic tools in cyanobacteria is the far-red light-inducible promoter *PchlFJSC1* from *Leptolyngbya* sp. JSC-1. *PchlFJSC1* activation is reversible under white light and has been used to control gene expression in the cyanobacterium *Chlorogloeopsis fritschii* PCC 9212 (Liu et al., 2023). *PchlFJSC1* function appears to rely on the expression of the far-red light photo-acclimation gene cluster *rfpABC*. Another example is the CcaS/CcaR system, which originates from the cyanobacterium *Synechocystis* sp. PCC 6803 and regulates gene expression in response to green/red light (Hirose et al., 2008) (Supplementary Figure S1). Wavelength-dependent control of green fluorescent protein (GFP) by CcaS/CcaR was successfully engineered in *Synechocystis* sp. PCC 6803 and *Synechococcus* NKBG 15041c (Abe et al., 2014; Badary et al., 2015). The CcaS/CcaR system has also been employed to generate a green light-activated cell lysis system, to allow cell surface display of proteins and to produce isobutanol and 3-methyl-1-butanol in *Synechocystis* sp. PCC 6803 (Miyake et al., 2014; Ferri et al., 2015; Kobayashi et al., 2022). Functionality has yet to be reported in other model cyanobacteria such as the fast-growing *Synechococcus* sp. PCC 7002, which can grow in saltwater and tolerate very high light intensities (Nomura et al., 2006). Furthermore, we lack detailed information on the kinetics and on the activity under a range of relevant light conditions.

A popular optogenetic system which, to our knowledge, has not yet been applied in cyanobacteria is the blue light-repressible YF1/FixJ system (Supplementary Figure S2). It was developed by replacing the heme-binding, oxygen-responsive PAS domain of the histidine kinase FixL of *Bradyrhizobium japonicum* with the blue light-sensitive LOV domain from *B*. *subtilis* YtvA (Möglich et al., 2009). This resulted in a chimeric protein in which histidine kinase activity is controlled by blue light rather than oxygen concentration. The YF1/FixJ system has been used to regulate gene expression in *E. coli*, *Pseudomonas aeruginosa* and *Vibrio natriegens* (Ohlendorf et al., 2012; Pu et al., 2018; Tschirhart et al., 2019).

In this study, we expressed the CcaS/CcaR and YF1/FixJ systems in *Synechococcus* sp. PCC 7002 and characterised their activity using Green Fluorescent Protein (GFP) fluorescence assays and qRT-PCR. We report the poor performance of the YF1/FixJ system and investigate why this might be. We show the functionality of the CcaS/CcaR system, and we provide characterisation of its activity under multiple different light conditions including constant light, light/dark cycles and alternating green/red light periods as well as kinetics of system activation and deactivation. Finally, we demonstrate the improved performance of a CcaS/CcaR system variant after targeted genetic modification of the *pCpcG2* output promoter. The presented results will facilitate future use of optogenetic tools for metabolic engineering in cyanobacteria.

## 2 Methods and materials

### 2.1 Culture and light treatments

*Synechococcus* sp. PCC 7002 was grown photo-autotrophically in modified artificial seawater (Medium A, Stevens Jr et al., 1973) supplemented with cycloheximide to a final concentration of 25 μg/mL to inhibit fungal contamination. Cryopreserved DMSO stocks were used to inoculate 20-mL pre-cultures which were grown inside glass tubes with a rubber stopper until an optical density at 730 nm (OD_730_) of 1-3 was reached. 0.5 mL of pre-culture diluted to OD_730_ = 1 was then added to 150 mL Medium A in 250 mL Bijou Bottles. Cultures were grown inside a Fitotron SGC-2 growth chamber (Weiss Technik) at 30 °C with a 16/8 h light/dark cycle. Humidified air was supplied via an aquarium pump and a flask containing distilled water. A Lambda 45 UV/VIS Spectrophotometer (PerkinElmer) was used for monitoring culture growth by measuring OD_730_.

The standard illumination was 150 μmol m^-2^ s^-1^ warm white LED light (Valoya Oy C65 NS12). To modify the wavelengths of light reaching the cultures light-transmitting filters were used (Supplementary Figure S3). The cultures were covered with the red-light transmitting filter Fire 019 (LEE Filters, 2023a) for Red and No Blue treatments or with the green light transmitting filter Dark Green 124 (LEE Filters, 2023b) for the Green treatment. The intensity of the white LED light source was adjusted to result in a final measured intensity of 30 μmol m^-2^ s^-1^.

For some experiments, custom-made 1-L MicroPharos photobioreactors (Xanthella, UK) were used. Temperature, light intensity and photoperiod in the reactors were controlled by the “Zeus II” control units with user-defined programmes. Light was provided by custom-made panels (‘rainbow tiles’) containing blue (λmax = 465 nm), cyan (λmax = 500 nm), green (λmax = 520 nm), orange (λmax = 590 nm) and red (λmax = 640 nm) LED lights. Aluminium foil was taped across the uncovered parts of the bioreactor vessel to prevent penetration of ambient light. As the rainbow tiles were attached to the vessel sides light intensity at the culture surface could not be measured. Instead, the light intensity of each treatment was measured at 5 cm from each tile inside the reactor. To grow *Synechococcus* sp. PCC 7002 cultures in the photobioreactors, 20-mL pre-cultures were grown to an OD_730_ of 1-3 inside the Fitotron growth cabinet. 3 mL of pre-culture diluted to OD_730_ = 1 was then added to 1 L of Medium A inside the bioreactor. 2 L of air per minute was injected using a vacuum pump which generated sufficient bubbling for culture mixing. Cultures were grown at 30 °C under different wavelength protocols.

### 2.2 Vector construction

All primers and vectors used in this study are listed in Supplementary Material S1, S2. Plasmid maps are available at: https://benchling.com/liamforbes7/f_/1MS6bd84-liam-frontiers-paper/. All restriction enzymes used were from New England Biolabs (NEB) (Hertfordshire, UK). PCR reactions were performed using NEB Phusion High-Fidelity DNA Polymerase and fragments were purified using QIAquick Gel Extraction Kit (QIAGEN, Venlo, Netherlands). Ligation reactions were performed with NEB T4 DNA Ligase using a 3:1 insert:vector ratio with an overnight incubation at 16 °C as per the manufacturer’s instructions. Gibson Assembly reactions were performed using gel-purified fragments and NEBuilder HiFi DNA Assembly Cloning Kit as per the manufacturer’s instructions. Plasmids assembled by restriction digest and ligation were transformed into One Shot Top10 *E. coli* (Invitrogen, California, USA) as per the manufacturer’s instructions. Successful construction of all plasmids was verified by restriction digest verification and sequencing (Source Bioscience, Cambridge, UK).

To test the activity of the YF1/FixJ system in *E. coli* we inserted GFP (GFPmut3b) into the pDusk vector (Ohlendorf et al., 2012; Addgene plasmid #43795; http://n2t.net/addgene:43795) after digestion with XbaI/XhoI.

To test the activity of the YF1/FixJ system in *Synechococcus* sp. PCC 7002 we generated pAQ1bBB-Blue. The pAQ1BB-GFP cyanobacterial transformation vector (Madsen et al., 2018) contains two flank sites that allow homologous recombination and integration into the native PAQ1 plasmid of *Synechococcus* sp. PCC 7002. GFP expression is driven by pCpc350, a truncated version of the strong cyanobacterial constitutive promoter pCpcB594 (Madsen et al., 2018). A DNA fragment containing the codon-optimised YF1/FixJ genes and the pFixK2 promoter were chemically synthesised (Integrated DNA Technologies, Leuven, Belgium) and inserted into pAQ1BB-GFP by ligation after digestion with EcoRI/XbaI to form pAQ1BB-Blue. To generate pAQ1BB-Blue-v2, the YF1/FixJ operon with a new RBS was amplified using from pAQ1BB-Blue using the YF1v2 F1 F and YF1v2 F1 R primers. The remainder of pAQ1BB-Blue was amplified using YF1v2 F2 F and YF1v2 F2 R and the two fragments were joined by Gibson Assembly to form pAQ1BB-Blue-v2.

To generate pAQ1BB-CcaSR-GFP, the CcaS promoter/gene was PCR amplified from pJT119b (Addgene plasmid #50551; http://n2t.net/addgene:50551) using primers CcaS F and CcaS R and inserted by ligation into the pGEM cloning vector after digestion with MluI/PstI to form pGEM-CcaS. The CcaR gene and pCpcG2 promoter were PCR amplified from pJT119b using primers CcaR F and CcaR R and ligated into pGEM-CcaS after digestion with HindIII/PstI to form pGEM-CcaS-CcaR. pGEM-CcaS-CcaR was digested with BsiWI/MluI to excise the CcaS-CcaR/pCpcG2 fragment, which was then ligated into pAQ1BB-GFP to form pAQ1BB-CcaSR-GFP (Supplementary Figure S4).

To generate pUC-172-GFP, pUC-172-10-GFP, pUC-172-35-GFP and pUC-172-35-10-GFP, primers were designed to perform PCR-based site-directed mutagenesis on pAQ1BB-CcaSR-GFP, which allowed amplification of the CcaS/CcaR/pCpcG2 DNA fragment from pAQ1BB-CcaSR-GFP with each of the desired modifications to the pCpcG2 promoter. The mutagenised fragments were assembled into the pAQ1BB plasmid backbone via Gibson Assembly. However, no *Synechococcus* sp. PCC 7002 transformants were obtained with any of these four constructs, likely due to the short homology arms of 250–400 bp. PCR reactions were then performed using primers CcaS SDM F1 and CcaS SDM R1 to amplify the mutagenised CcaS/CcaR/pCpcG2 DNA fragments from the pAQ1BB plasmid backbones. The amplified fragments were then assembled into the pUC-Spec plasmid which contained 1000-bp homology arms using Gibson Assembly.

### 2.3 Cyanobacterial transformation

For the natural transformation of *Synechococcus* sp. PCC 7002, 1.5 mL of culture grown to OD_730_ of ∼1 was incubated with 1–20 µg of DNA for 3 days under 20 μmol m^-2^ s^-1^ white light at 30 °C with very slow bubbling. After 3 days, the cultures were pelleted at 13,000 x rpm for 2 min, resuspended in 100 µL of supernatant, and spread on plates containing Medium A and 50 μg/mL spectinomycin for selection. Plates were then incubated under 30 μmol m^-2^ s^-1^ white light at 30 °C for 7–10 days. Successful transformants were verified using colony PCR. Colonies were continuously re-streaked on Medium A plates of successively higher antibiotic concentration until full segregation was achieved where possible. Colonies were then used to inoculate a 20-mL liquid culture.

### 2.4 GFP reporter assay

GFP fluorescence of 1-mL culture samples was determined with a Perkin Elmer LS 55 Luminescence Spectrophotometer using excitation and emission wavelengths of 480 nm and 514 nm, respectively and a 10 nm path length. Measured GFP fluorescence was normalised to OD_730_ for *Synechococcus* sp. PCC 7002 and OD_600_ for *E. coli* as proxies for cell number. For measurement of GFP expression in *E. coli*, 1-mL samples were taken and pelleted at 13,000 x rpm for 2 min before being washed once with 1 X phosphate-buffered saline (PBS) and resuspended in 1-mL PBS to remove LB media which produces high background signal. GFP fluorescence was then measured in 1 mL of the resuspended culture.

### 2.5 qRT-PCR analysis of gene expression

Total RNA was extracted using the RNeasy Mini Kit (QIAGEN, Venlo, Netherlands) with on-column DNase digestion. Resuspended cell pellets were lysed with 0.5 g of acid-washed glass beads (425–600 µm diameter) using the TissueLyser (QIAGEN, Venlo, Netherlands) at 30 Hz for 5 min. The RNA was then purified using the RNeasy Mini Kit as per the manufacturer’s instructions. RNA concentration and purity were determined using the NanoDrop One spectrophotometer (Thermo Fisher Scientific, Rochester, USA).

For quantitative real-time PCR (qRT-PCR), 1 µg total RNA was transcribed into cDNA using the QuantiTect Reverse Transcription Kit (QIAGEN, Venlo, Netherlands) as per the manufacturer’s instructions. Primers were designed to amplify 80–150 bp regions of cDNA target genes with an annealing temperature of 60 °C. Standard curves were generated by serial dilution of purified PCR product amplified from cDNA. The standard curve was used to convert cycle (Cq) values into RNA concentrations, which were then normalised to the reference gene *SYNPCC7002_A1356*. *SYNPCC7002_A1356* was chosen as a reference gene after measuring the expression of several candidate genes and determining which gene showed the least variation across the experimental conditions using RefFinder (Supplementary Figure S5) (Xie et al., 2023). qRT-PCR reactions were performed using the StepOnePlus Real-Time PCR System (Life Technologies, Rochester, USA) and visualised with Brilliant III Ultra-Fast SYBR Green QPCR Master Mix (Agilent, Santa Clara, USA). Post-assay melt curve analysis confirmed the absence of primer dimers. Negative controls lacking cDNA template were included on each plate. Three biological replicates were used per condition and technical replication of each reaction was performed in triplicate. Primers used are shown in Supplementary Material S1.

## 3 Results

### 3.1 The YF1/FixJ system demonstrates minimal activity in *Synechococcus* sp. PCC 7002

To confirm functionality of the YF1 system in *E. coli*, we transformed *E. coli* with pDusk-GFP allowing expression of YF1 and FixJ as a bicistronic operon from the *E. coli* constitutive promoter LacIq (Ohlendorf et al., 2012) and of GFP from pFixK2. The presence of the correct DNA sequence in clones growing on selective media was confirmed by restriction digest analysis and sequencing. A successfully transformed clone was used to inoculate 150-mL cultures. These were grown to OD_600_ of ∼0.3 under 100 μmol m^-2^ s^-1^ white LED light before being wrapped in foil (Dark) or left uncovered (Light). GFP fluorescence was measured 3 h after the respective light treatments, normalised to OD_600_ and the background fluorescence of cultures expressing the empty pDusk vector was subtracted. After 3 h, GFP fluorescence was 45-fold higher in cultures incubated in darkness than those incubated under white light (Supplementary Figure S6). This confirmed that the original YF1 system is functional in *E. coli*.

For expression in *Synechococcus* sp. PCC 7002 we generated pAQ1BB-Blue, allowing expression of the YF1/FixJ operon from a medium-high strength cyanobacterial constitutive promoter and of GFP from pFixK2. *Synechococcus* sp. PCC 7002 cells were transformed with pAQ1BB-Blue and grown on selective media. The presence of the correct DNA in selected clones was confirmed by colony PCR and sequencing. As a control, *Synechococcus* sp. PCC 7002 were also transformed with empty pAQ1BB vector (pAQ1BB). 150-mL *Synechococcus* sp. PCC 7002 cultures expressing pAQ1BB-Blue or pAQ1BB were grown for 4 days to OD_730_ of ∼1 under white LED light. Cultures were then either covered with the red-light transmitting filter (No Blue Light, No BL) or left uncovered (BL). GFP fluorescence and OD_730_ were measured at several time points.

**FIGURE 1:**
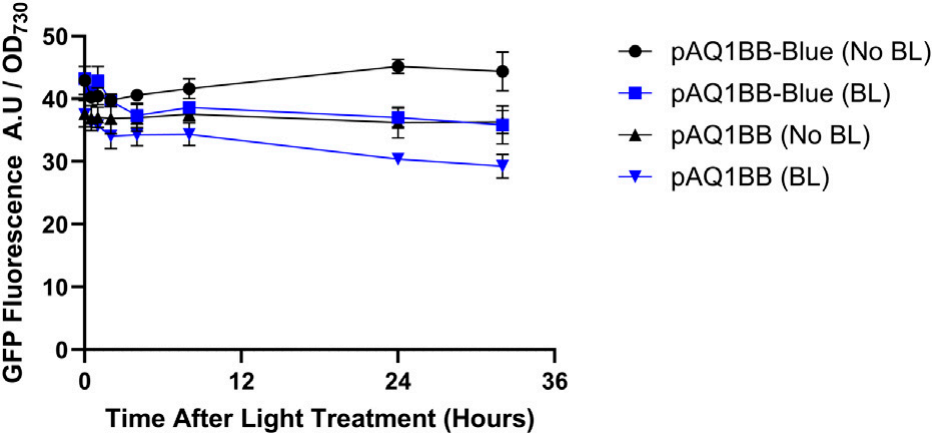
GFP-fluorescence driven by the YF1/FixJ system in *Synechococcus* sp. PCC 7002 in the presence and absence of blue light (BL). 150-mL *Synechococcus* sp. PCC 7002 cultures expressing either GFP under control of the YF1/FixJ system (pAQ1BB-Blue) or empty pAQ1BB vector (pAQ1BB) were grown to OD_730_ of ∼1 under 150 µmol white LED light. The cultures were then either covered with a red-light transmitting filter (No BL) (black symbols) or left uncovered (BL) (blue symbols). GFP fluorescence was measured and normalised to OD_730_. Data are means ± S.E.M of three independent cultures. No significant differences at *p* < 0.05 were determined by unpaired t-test.

No significant differences in GFP fluorescence were observed between the two light treatments across most of the time points (Figure 1). After 32 h, the No BL pAQ1BB-Blue cultures produced only slightly higher fluorescence than the BL pAQ1BB-Blue cultures, with a *p*-value of 0.06. A similar difference between the BL and No BL treatments was also observed in the empty vector control. Under both light treatments, pAQ1BB-Blue and the pAQ1BB cultures showed similar levels of fluorescence. Given the 16/8 h light/dark cycle, the No BL pAQ1BB-Blue cultures would have been in the ‘induced’ state for 32 h. These results suggest that the YF1/FixJ system is not functional in *Synechococcus* sp. PCC 7002.

### 3.2 Troubleshooting steps fail to improve YF1/FixJ system performance in *Synechococcus* sp. PCC 7002

**FIGURE 2:**
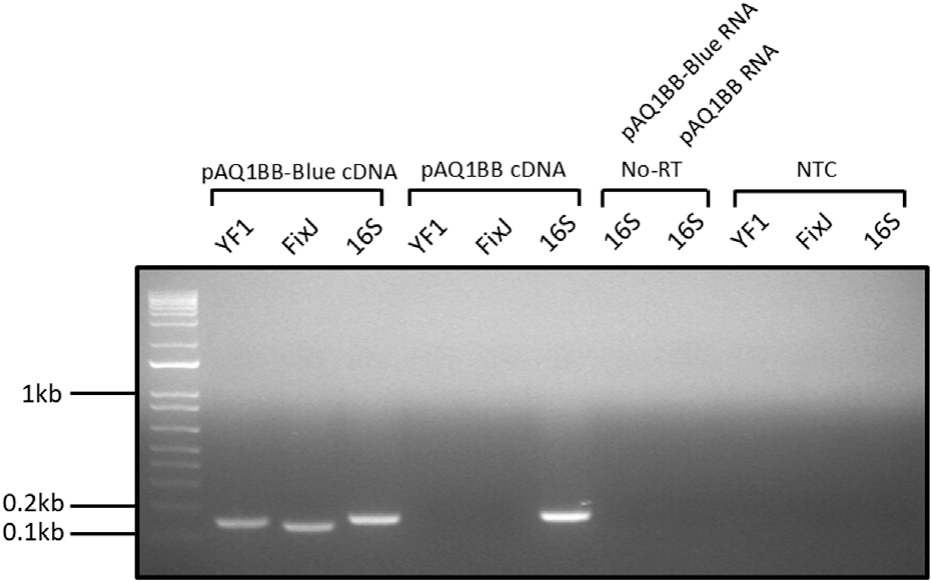
Gel image of YF1 and FixJ RT-PCR reactions from pAQ1BB-Blue culture cDNA. pAQ1BB-Blue and pAQ1BB cultures were grown to OD_730_ of ∼1 under standard conditions, RNA was extracted, and cDNA was synthesised from 0.5 µg RNA. 0.5 µL undiluted cDNA was then used in 25 µL PCR reactions containing either YF1, FixJ, or 16S rRNA primers. Reactions were run for 30 cycles and visualised on a 1.5% agarose gel stained with ethidium bromide. No-RT = no reverse transcriptase, NTC = no template control.

Poor performance of the YF1/FixJ system in *Synechococcus* sp. PCC 7002 could be due to a lack of YF1 and FixJ gene expression. To test this, RNA was extracted from pAQ1BB-Blue and pAQ1BB cultures and used to produce cDNA, which was used as a template for RT-PCR analysis and visualised by agarose gel electrophoresis. Primers were designed to amplify short 100–200 bp regions of the YF1 and FixJ genes and to amplify the constitutively expressed 16S rRNA as a control. Clear bands were observed in the pAQ1BB-Blue cDNA YF1 and FixJ lanes (Figure 2). The absence of bands in the no-template control (NTC) and pAQ1BB samples highlighted that amplification in the pAQ1BB-Blue cDNA samples was not due to primer dimers or non-specific amplification from the *Synechococcus* sp. PCC 7002 genome. RNA samples used as a template for cDNA synthesis in the absence of reverse transcriptase (No-RT) also demonstrated that any amplification observed in the pAQ1BB-Blue cDNA samples did not arise from contaminating gDNA. The results show that the YF1 and FixJ genes were expressed in the pAQ1BB-Blue cells.

Finally, we tested whether the performance of the YF1 system was limited by the efficiency of YF1 or FixJ translation. The Salis RBS calculator v2.2 was used to calculate a predicted translation initiation rate of 100 arbitrary units (A.U) at the YF1 and 1000 A.U FixJ start codons (Reis and Salis, 2020). Subsequently, a new YF1 RBS was designed using the RBS calculator with a predicted translation initiation rate of 2000 A.U. This stronger RBS was then inserted directly upstream of YF1 in the pAQ1BB-Blue construct, replacing the original RBS. This new construct was designated as pAQ1BB-Blue v2. *Synechococcus* sp. PCC 7002 were transformed with pAQ1BB-Blue v2 and grown on selective media. The presence of the correct DNA in selected clones was confirmed by restriction digest analysis and sequencing. 150-mL *Synechococcus* sp. PCC 7002 cultures expressing pAQ1BB-Blue v2 or empty vector (pAQ1BB) were grown to OD_730_ of ∼1 under white light. Cultures were then either covered with foil (Dark) or left uncovered (Light) and GFP fluorescence and OD_730_ were measured at several time points across 48 h.

**FIGURE 3:**
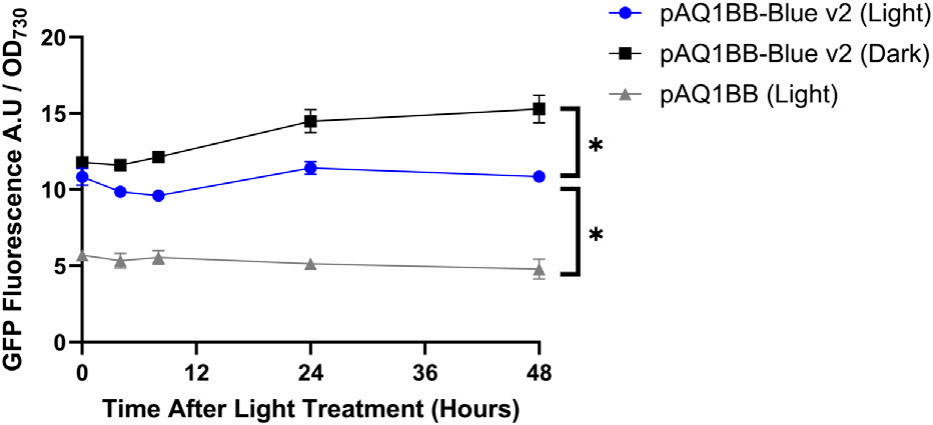
YF1-v2 system activity in Synechococcus sp. PCC 7002 in blue light and darkness. 150-mL *Synechococcus* sp. PCC 7002 cultures expressing either GFP under control of the YF1/FixJ system (pAQ1BB-Blue-v2) (blue and black symbols) or empty pAQ1BB vector (pAQ1BB) (grey symbols) were grown to OD_730_ of ∼1 under 150 µmol white LED light. The cultures were then either covered with foil (Dark) or left uncovered (Light). As cultures were grown under a 16/8 h light/dark cycle, the cultures were in the dark between 16 and 24 h after the light treatment began. GFP fluorescence was measured and normalised to OD_730_. Data are means ± S.E.M of three independent cultures. Significant differences at *p* < 0.05 as determined by unpaired t-test are shown by asterisk.

After 48 h, GFP fluorescence in pAQ1BB-Blue v2 cultures incubated in darkness was 1.5-fold higher than in 1BB-Blue v2 cultures incubated in light (Figure 3). Compared to the pAQ1BB cultures, GFP fluorescence in pAQ1BB-Blue v2 cultures was 3-fold higher in the light, and 2-fold higher in the dark. The differences were statistically significant. While these experiments showed some blue-dependent GFP production mediated by pAQ1BB-Blue v2 the result was only marginally better than for the original pAQ1BB-Blue (Figure 1). A low translation initiation rate is therefore not the main reason for poor performance of the YF1 system in *Synechococcus* sp. PCC 7002.

In summary, our experiments tested several possible explanations for the poor performance of the YF1 system in *Synechococcus* sp. PCC 7002 but ultimately failed to identify the underlying reason for this.

### 3.3 Expression of the CcaS/CcaR system does not affect *Synechococcus* sp. PCC 7002 growth rate

To test the performance of the green/red light responsive CcaS/CcaR system, the pAQ1BB-CcaSR-GFP plasmid was cloned and expressed in *Synechococcus* sp. PCC 7002. GFP was inserted directly downstream of the green/red light-responsive pCpcG2 promoter, with CcaS and CcaR expressed from their native promoters (Supplementary Figure S4). In this plasmid the CcaS-CcaR-pCpcG2-GFP fragment was placed between two flank sites, allowing homologous recombination and integration into the high-copy number *Synechococcus* sp. PCC 7002 endogenous plasmid pAQ1. After sequence verification, a transformed clone was used as inoculate for 1-L cultures in Xanthella photobioreactors. The cultures were illuminated with an equivalent intensity of red or green light provided by narrowband LEDs. In separate experiments, 150-mL cultures of both wildtype *Synechococcus* sp. PCC 7002 and *Synechococcus* sp. PCC 7002 expressing pAQ1BB-CcaSR-GFP were grown in 250-mL Bijou bottles under either red or green light as provided by white light LEDs with red or green filters (see Materials and Methods for details).

**FIGURE 4:**
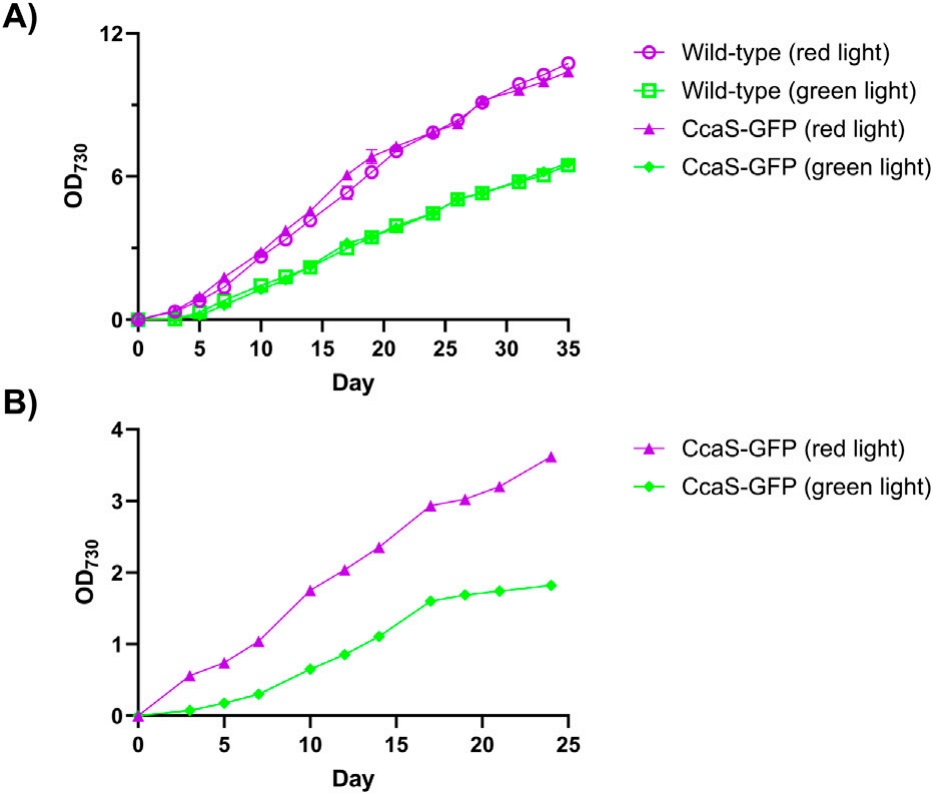
Growth of *Synechococcus* sp. PCC 7002 expressing the CcaS/CcaR system. Culture density (OD_730_) of wildtype *Synechococcus* sp. PCC 7002 (open symbols) or *Synechococcus* sp. PCC 7002 expressing pAQ1BB-CcaSR-GFP (CcaS-GFP, filled symbols). **(A)** 150-mL cultures were grown in 250-mL Bijou bottles under illumination with 30 μmol m^-2^ s^-1^ red light (purple symbols) or green light (green symbols) provided by white LED light combined with the red or green light-transmitting filters. **(B)** *Synechococcus* sp. PCC 7002 cultures expressing pAQ1BB-CcaSR-GFP were grown in 1-L photobioreactors and illuminated with an equivalent intensity of red or green narrowband LED light (purple and green symbols). All cultures were grown under 16 h/8 h light/dark cycles. Data are means ± S.E.M of three independent cultures.

OD_730_ measurements in the 150-mL cultures showed that growth curves of *Synechococcus* sp. PCC 7002 expressing pAQ1BB-CcaSR-GFP were similar to those of wildtype *Synechococcus* sp. PCC 7002 under both red and green light (Figure 4A). Thus, the expression of the CcaS/CcaR system did not impact the growth of *Synechococcus* sp. PCC 7002. Culture growth was generally faster under red light than under green light as would be expected given the preferential wavelength usage for photosynthesis (Figures 4A,B).

### 3.4 Green light increases CcaS/CcaR system activity whereas red light does not

To test and characterise the CcaS/CcaR system in *Synechococcus* sp. PCC 7002, GFP fluorescence was measured under different light treatments. *Synechococcus* sp. PCC 7002 cultures expressing pAQ1BB-CcaSR-GFP were grown in 1-L photobioreactors under constant red light for 4 days to OD_730_ of ∼1. The cultures were then given different light treatments consisting of either red light only, green light only, an equivalent intensity of combined red and green light (Red + Green), green light of 2.5-fold increased intensity, or darkness (for a detailed description of the light treatments see Materials and Methods). Samples were harvested at the indicated time points before GFP fluorescence was measured and normalized to OD_730_ as a proxy of cell number.

**FIGURE 5:**
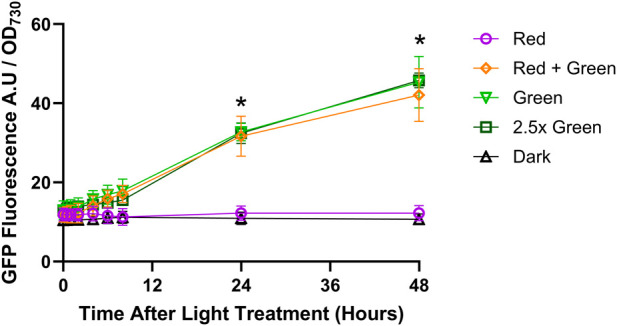
CcaS/CcaR system activity under constant light conditions. 1-L cultures of *Synechococcus* sp. PCC 7002 expressing pAQ1BB-CcaSR-GFP were grown for 4 days in photobioreactors under constant red light. Cultures were then illuminated with the following light treatments: Red = 20 μmol m^-2^ s^-1^ red LED (purple symbols); Red + Green = 20 μmol m^-2^ s^-1^ red LED +20 μmol m^-2^ s^-1^ green LED (orange symbols); Green = 20 μmol m^-2^ s^-1^ green LED (pale green symbols); 2.5x green = 50 μmol m^-2^ s^-1^ green LED (dark green symbols); Dark = darkness (black symbols). GFP fluorescence was measured, normalised to OD_730_ and background fluorescence measured in cells expressing empty pAQ1BB vector was subtracted. Data are means ± S.E.M of three independent cultures. Significance at *p* < 0.05 between Green *versus* Red at selected time points as determined by paired t-tests are shown by asterisks.

Under red light illumination, GFP fluorescence remained constant over 48 h but was not zero (Figure 5). GFP fluorescence also remained constant in the dark-incubated cultures, with similar values as measured in the red light treatment. Under illumination with green light only or with red plus green light, GFP fluorescence rose steadily over the 48 h. At 24 h and 48 h, GFP fluorescence was roughly 2-fold and 3.5-fold higher, respectively, than under red light only. Increasing the green light intensity 2.5x green) did not further increase GFP fluorescence. Collectively, this data shows that green light either alone or added to red light, increases the activity of the CcaS/CcaR system. GFP fluorescence remained low, but above zero, under red light and in darkness, indicating a constant background transcription even when CcaS is in the dark state.

### 3.5 Green light causes rapid activation of the CcaS/CcaR system at the transcriptional level

**FIGURE 6:**
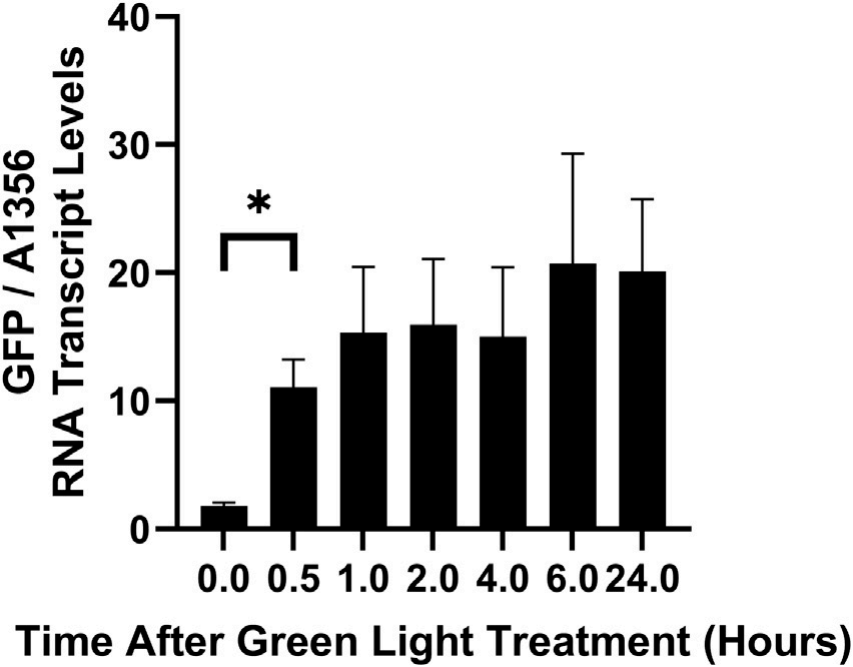
The rate of CcaS/CcaR system activation under green light as measured by qRT-PCR. 1-L cultures of *Synechococcus* sp. PCC 7002 expressing pAQ1BB-CcaSR-GFP were grown for 4 days in photobioreactors under constant red LED light. Cultures were then illuminated for 24 h with green light only and samples were harvested for RNA extraction at the indicated timepoints. GFP RNA levels were measured using qRT-PCR and normalised to *SYNPCC7002_A1356* RNA levels. Data are means ± S.E.M of three independent cultures. Significance at *p* < 0.05 was calculated using two-tailed paired t-tests.

The slow increase in GFP fluorescence under green light (Figure 5) could be due to slow protein production. To better understand the kinetics of CcaS activation without the confounding effects of GFP translation and protein folding, we measured GFP RNA transcript levels using qRT-PCR. Cultures expressing pAQ1BB-CcaSR-GFP were grown in photobioreactors under constant red light for 4 days until they reached OD_730_ of ∼1. Cultures were then illuminated with green light only and GFP transcript levels were measured at several time points. Whilst a fully segregated pAQ1BB-CcaSR-GFP strain was not achieved, it was demonstrated that any differences in GFP expression between light conditions and time points was not due to natural fluctuations in endogenous pAQ1 plasmid copy number or the ratio of GFP transgene to pAQ1 plasmid (Supplementary Figure S7). GFP RNA levels increased 6-fold after 30 min of green light illumination (Figure 6). Mean GFP transcript levels appeared to increase slightly further to reach a maximum at 6 h but due to variation, any differences were not significant. This data shows that the CcaS/CcaR system is activated rapidly in response to green light.

### 3.6 CcaS/CcaR system activity decreases significantly after several hours of red light

After demonstrating rapid activation of the CcaS/CcaR system under green light, we tested whether GFP transcript levels decreased in response to red light. Cultures expressing pAQ1BB-CcaSR-GFP were grown in photobioreactors under constant red light for 4 days until they reached OD_730_ of ∼1. The cultures were then illuminated with green light for 24 h to activate the CcaS/CcaR system, before switching back to red light and measuring GFP RNA transcript levels using qRT-PCR.

**FIGURE 7:**
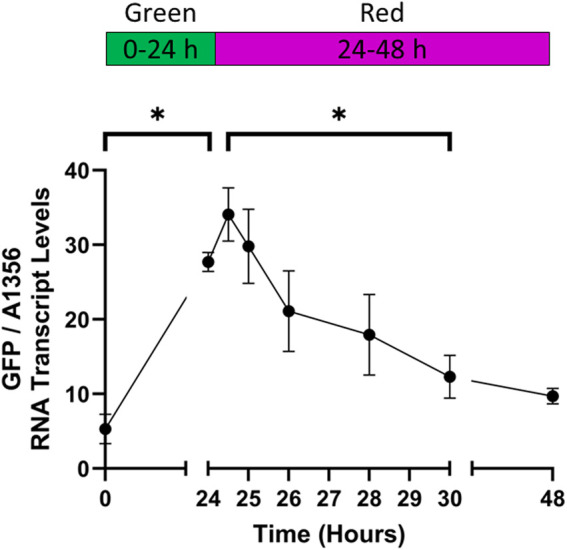
The rate of CcaS/CcaR system deactivation under red light. 1-L cultures of *Synechococcus* sp. PCC 7002 expressing pAQ1BB-CcaSR-GFP were grown for 4 days in photobioreactors under constant red LED light. Illumination was switched to green light for 24 h, before switching back to red light illumination for 24 h. RNA was extracted from samples harvested at the indicated time points, GFP RNA transcript was measured by qRT-PCR and normalised to *SYNPCC7002_A1356* RNA transcript levels. Data are means ± S.E.M of three independent cultures. Significance at *p* < 0.05 was determined using two-tailed paired t-tests.

After 24 h of green light illumination, GFP RNA levels were 5 times higher than at time point 0 (Figure 7). Upon switching back to red light only, GFP RNA levels continued to increase for another hour before starting to decrease with statistically significant differences measured after 6 h (30 h time point). A small further reduction was seen in GFP RNA transcript after 24 h of red light (48 h time point). At this point, GFP RNA transcript was still around 2-fold higher than at the 0-hour timepoint although the difference was not significant. In summary, by measuring GFP RNA transcript levels, we could show effective red-light deactivation of CcaS/CcaR. The recorded rates of de-/activation are likely to be limited by the speed of RNA degradation and transcription rather than de-/phosphorylation of the photoreceptor. Some variation in the kinetics would therefore be expected depending on target transcript albeit probably less than at protein level.

### 3.7 The CcaS/CcaR system can be reversibly and repeatedly activated by alternating green and red light periods

To enable the use of dynamic metabolic pathway control, an optogenetic system must be capable of repeated reversible activation and deactivation in response to different light wavelengths. This ability has yet to be demonstrated for the CcaS/CcaR system. To test the response of the CcaS/CcaR system to alternating periods of green and red light, cultures expressing pAQ1BB-CcaSR-GFP were grown in photobioreactors for 4 days under constant red light. The cultures were then illuminated with alternating 24-hour periods of green light or red light and GFP transcript levels were measured at several time points using qRT-PCR.

**FIGURE 8:**
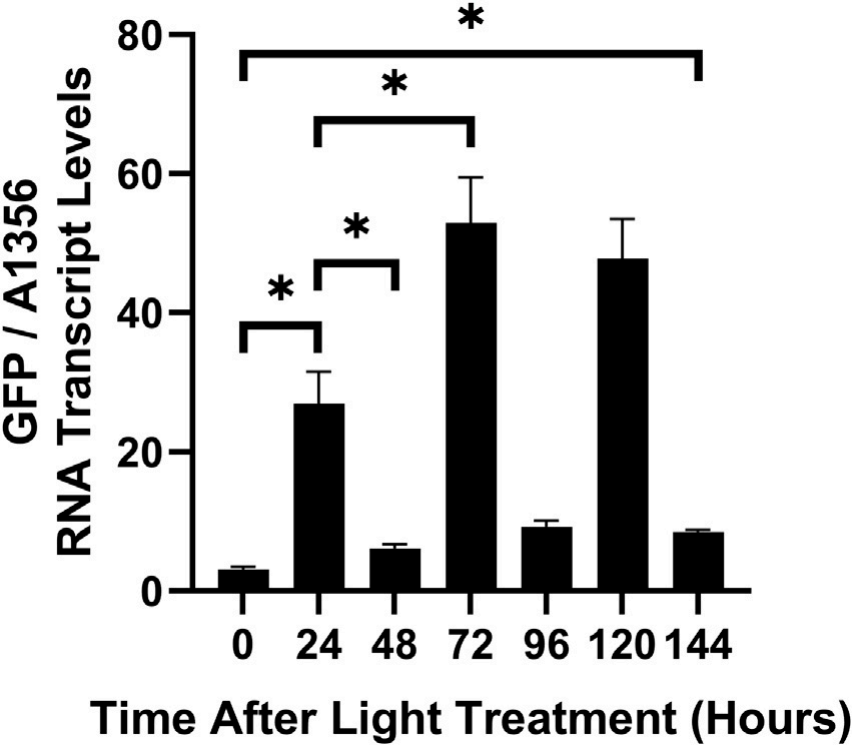
CcaS/CcaR system activity during alternating periods of red or green light. 1-L cultures of *Synechococcus* sp. PCC 7002 expressing pAQ1BB-CcaSR-GFP were grown for 4 days in photobioreactors under constant red LED light. Cultures were then illuminated with alternating 24-h periods of green or red light. RNA was extracted from samples harvested at the indicated time points, GFP RNA was measured by qRT-PCR and normalised to *SYNPCC7002_A1356* RNA levels. Data are means ± S.E.M. of three independent cultures. Significance at *p* < 0.05 was determined using two-tailed paired t-tests.

As shown in Figure 8, the CcaS/CcaR system up- and downregulated transcript levels of the target gene over repeated green/red light cycles. GFP RNA levels rose significantly over 24 h of green light (Figure 8). GFP transcript levels then decreased in the subsequent 24 h, although remaining higher than at the 0-hour timepoint (*p* = 0.052). The second 24-hour period of green light resulted in GFP transcript levels that were close to double that of the first 24-hour period of green light, a statistically difference. GFP transcript levels decreased after the second 24-hour period of red light but remained slightly higher than those measured after the first 24-hour period of red light. After the third 24-hour period of green light, GFP transcript levels increased to a similar level as the second 24-hour period of green light (no statistically significant difference). Finally, the third 24-hour period of red light resulted in a similar decrease in GFP transcript to that of the second 24-hour period of red light. At the end of the third 24-hour light period (144 h), GFP RNA transcript was significantly higher than at the 0-hour timepoint. This data confirms that the CcaS/CcaR system can be repeatedly activated and deactivated under alternating 24-hour periods of green and red light. The transcriptional response increases over the first two cycles, indicating a “primed state” of either photoreceptor or transcriptional machinery.

### 3.8 CcaS/CcaR system functions under light-dark cycles, with a slow decrease of GFP RNA transcript during dark cycles

As photosystem II suffers damage under lengthy periods of constant illumination, cyanobacteria are typically grown under light/dark cycles. This allows photosystem repair during darkness, and sustained growth (Nixon et al., 2010). However, previous characterisation of CcaS/CcaR activity in cyanobacteria was performed under constant light conditions. To test the effect of dark periods on CcaS/CcaR system activity, pAQ1BB-CcaSR-GFP cultures were grown in photobioreactors under red light using a 16 h/8 h light/dark photoperiod for 5 days until cultures reached OD_730_ of ∼1. Illumination was then switched to green light only whilst maintaining the 16 h/8 h light/dark photoperiod and GFP RNA transcript levels were measured at various time points using qRT-PCR.

**FIGURE 9:**
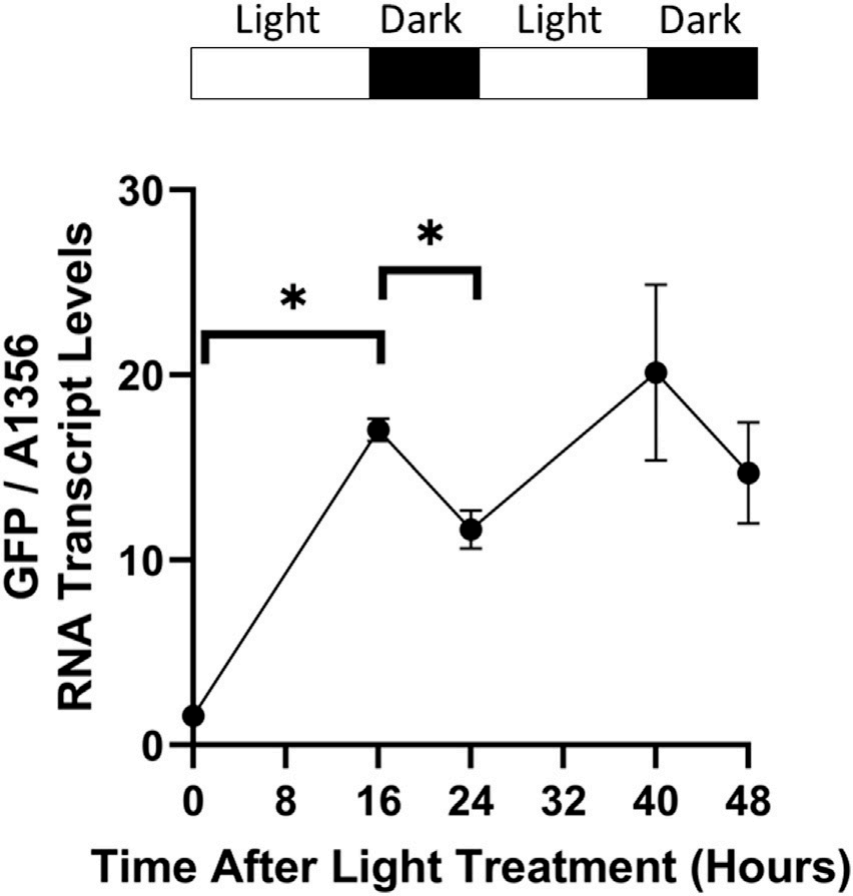
CcaS/CcaR system activity under light/dark cycles. 1-L cultures of *Synechococcus* sp. PCC 7002 expressing pAQ1BB-CcaSR-GFP were grown for 5 days in photobioreactors under red light using a 16 h/8 h light/dark cycle. Whilst maintaining the same light/dark photoperiod, cultures were then illuminated with green light during the subsequent 48 h. RNA was extracted from samples harvested at the indicated time points, GFP RNA transcript was measured by qRT-PCR and normalised to *SYNPCC7002_A1356* RNA transcript levels. Data are means ± S.E.M of three independent cultures. Significance at *p* < 0.05 was determined using a two-tailed paired t-test.

GFP RNA transcript levels increased during the first 16-hour green light period before decreasing by around 30% in the first dark period (Figure 9). GFP transcript levels increased again during the second light cycle to a slightly higher level than at the end of the first green light period. However, the relative increase was lower during this second light cycle and the difference in GFP RNA transcript levels at the end of each green light period was not statistically significant. GFP transcript levels then decreased again in the second dark period at a similar rate to the first dark cycle. The results show that an 8-hour dark period is not sufficient to decrease GFP RNA transcript levels back to baseline.

### 3.9 Genetic modification of the pCpcG2 output promoter increases CcaS/CcaR system activity under green light and improves dynamic range

**FIGURE 10:**
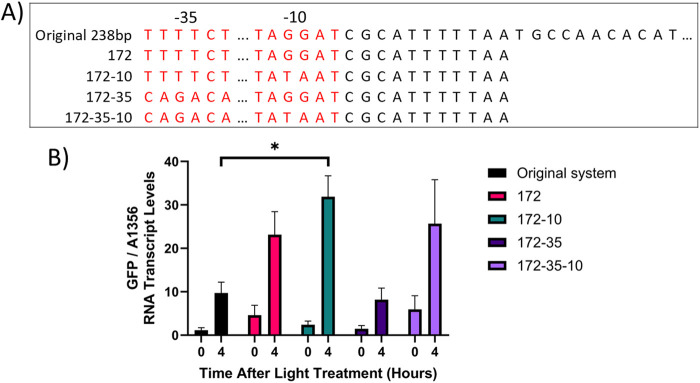
CcaS/CcaR system activity after modification of pCpcG2. **(A)** Schematic showing the sequence of pCpcG2 in the following constructs: pAQ1BB-CcaSR-GFP (Original system), pUC-172-GFP (172), pUC-172-10-GFP (172-10), pUC-172-35-GFP (172-35) and pUC-172-35-10-GFP (172-35-10). The −35 and −10 hexamers are highlighted in red. Dots indicate constant regions of sequence omitted for simplicity. **(B)** 150-mL cultures of *Synechococcus* sp. PCC 7002 cultures expressing the above constructs were grown for 4 days under illumination with red light as provided by red light transmitting filters combined with warm white LED lights. The cultures were then switched to green light as provided by green light transmitting filters. RNA was extracted from samples harvested at the indicated time points, GFP RNA levels were measured by qRT-PCR and normalised to *SYNPCC7002_A1356*. Data are means ± S.E.M of three independent cultures. Significance at *p* < 0.05 was determined using two-tailed paired t-tests.

Two factors which affect the utility of an optogenetic system are the dynamic range, i.e., the fold-increase in system activity between the ON/OFF states, and the maximal level of transcriptional output achieved in the ON state. The system component which was deemed most likely to affect these factors was the pCpcG2 output promoter. In *E. coli* truncation of pCpcG2 reduced the basal activity of the promoter in red light, presumably due to the removal of a predicted weak constitutive promoter present (Schmidl et al., 2014). In green light, modifying the −35 and −10 hexamers of pCpcG2 to match those of the strong *Synechococcus* sp. PCC 7002 constitutive promoter pCpct223 could be a means to increase the maximal activity of the CcaS/CcaR system (Markley et al., 2015). We used site-directed mutagenesis (SDM) to generate the constructs pUC-172-GFP, pUC-172-10-GFP, pUC-172-35-GFP and pUC-172-35-10-GFP, in which expression of GFP was controlled by the modified pCpcG2 promoters (Figure 10). All constructs contained the 172 bp truncated pCpcG2 promoter combined with various combinations of −10 and −35 hexamers from the pCpct223 strong promoter. They were inserted into a pUC19 backbone plasmid which improved cloning efficiency. Furthermore, the homology arms for recombination into pAQ1 were extended from 250 to 350 bp to 1,000 bp to increase transformation efficiency and achieve fully segregated strains (Supplementary Figure S8). 150-mL cultures expressing the constructs were grown under red light using a 16 h/8 h light/dark photoperiod for 5 days until cultures reached OD_730_ ∼1. The cultures were then switched to illumination with green light for 4 h and GFP transcript levels were measured using qRT-PCR.

The original CcaS/CcaR system demonstrated a dynamic range of 8.5-fold after 4 h of green light (Figure 10), which was similar to previous experiments (Figures 6, 8). In cultures expressing pUC-172, which contained a truncated 172 bp version of the original 232 bp pCpcG2 promoter, GFP RNA transcript levels were higher under both red light and green light than the original system but the dynamic range was only 5-fold. In cultures expressing pUC-172-10, which contained the truncated pCpcG2 with a modified −10 hexamer, GFP RNA transcript levels under green light were 3-fold higher than in the original system, resulting in an improved dynamic range of 13.3-fold. In cultures expressing pUC-172-35, which contained truncated pCpcG2 with a modified −35 hexamer, GFP transcript levels under green light were lower than that of the original system, with a reduced dynamic range of 5.5-fold. In cultures expressing pUC-172-35-10, which contained truncated pCpcG2 with both −35 and −10 hexamers modified, GFP RNA transcript levels under both red and green light were higher than the original system, resulting in the lowest dynamic range of 4.3-fold. The data showed that the combination of pCpcG2 truncation and modification of the −10 hexamer (pUC-172-10-GFP) results in a CcaS/CcaR system variant with improved properties compared to the original.

## 4 Discussion

### 4.1 Poor performance of the YF1/FixJ system

When the YF1/FixJ system was expressed in *E. coli*, a 46-fold increase in GFP fluorescence was observed in the dark-incubated cultures *versus* the light-incubated cultures (Supplementary Figure S5), confirming that the construct used in this study was functional in *E. coli* and showed similar blue light sensitivity as previously reported (70-fold increase, Möglich et al., 2009). However, subsequent results showed that the YF1/FixJ system did not function when expressed in *Synechococcus* sp. PCC 7002. No significant differences in GFP fluorescence were observed between pAQ1BB-Blue cultures in the presence or absence of blue light, with fluorescence levels barely above those measured in the 1BB empty vector control (Figure 1). The slight differences in fluorescence observed between pAQ1BB-Blue/pAQ1BB-Blue-v2 and the pAQ1BB cultures towards the end of the time course may be due to chromatic acclimation in response to differing light conditions rather than increased expression of GFP. RT-PCR analysis confirmed that both the YF1 and FixJ genes were well expressed in *Synechococcus* sp. PCC 7002 (Figure 2) with gel bands only slightly fainter than those of 16S rRNA. Efforts to increase the translation rate by using a new RBS with a predicted 20-fold higher strength only had a marginally positive effect on system performance (Figure 3). For context, the predicted translation initiation rate of GFP in the pAQ1BB-CcaS-CcaR construct (Figure 5) was around 5000 A.U, whilst the newly designed YF1/FixJ RBS was 2000 A.U.

There are several other plausible hypotheses which could explain the poor performance of YF1 in *Synechococcus* sp. PCC 7002. The first is that in the intracellular environment of *Synechococcus* sp. PCC 7002, YF1 is unable to phosphorylate FixJ. It is also possible that YF1 can phosphorylate FixJ, but that an unknown endogenous protein phosphatase dephosphorylates FixJ at a much faster rate. Another hypothesis is that an endogenous repressor protein could bind to pFixK2 in *Synechococcus* sp. PCC 7002, blocking access to the phosphorylated FixJ. Alternatively, a repressor protein or allosteric inhibitor may bind directly to FixJ, preventing its interaction with pFixK2. It is also possible that an additional unknown protein, such as a transcriptional activator, is necessary for the initiation of transcription from pFixK2. This protein could be present in species in which the YF1/FixJ system has been successfully deployed such as *E. coli* and *V. natriegens* but absent from *Synechococcus* sp. PCC 7002. Future work should test these hypotheses in an attempt to improve the transferability of the YF1/FixJ system to cyanobacteria.

### 4.2 Comparison of CcaS/CcaR system performance in *Synechococcus* sp. PCC 7002 and other organisms

The CcaS/CcaR system originates from a cyanobacterium (*Synechocystis* PCC 6803; Hirose et al., 2008) and functionality has already been shown in another cyanobacterial species (*Synechococcus* NKBG 15041c; Abe et al., 2014; Badary et al., 2015). However, wider transferability between different strains still needs to be tested. Furthermore, essential kinetic properties remain to be characterised. Expressing the CcaS/CcaR system in *Synechococcus* sp. PCC 7002 we observed a 3.5-fold increase in GFP fluorescence after 48 h of constant green light or red plus green light (Figure 5). Albeit significant, this was less than the 10 to 11-fold increase reported for *Synechocystis* sp. PCC 6803, after 39 h of green light alone and with red light (Abe et al., 2014) or the 7-8-fold increase reported for *Synechococcus* NKBG 15041c after 48 h of green plus red light (Badary et al., 2015). Differences between the reported results could be due to differences in pigment composition between species affecting the amount of light absorbed, as well as differences in the amount of CcaS and CcaR protein produced. Measuring transcript rather than protein provides a more accurate reflection of the dynamics of the optogenetic system. In *Synechococcus* sp. PCC 7002, we found a 6-fold increase in GFP RNA transcript levels within 30 min of green light illumination (Figure 6), which was much more rapid than the increase in GFP fluorescence (Figure 5).

There is only limited information on deactivation kinetics of optogenetic systems in cyanobacteria. Determination of both activation and de-activation kinetics was previously attempted for the far-red light-activated promoter *P*_*chlF*JSC1_ from the cyanobacterium *Leptolyngbya* sp. JSC-1 (Liu et al., 2023). When used to drive the expression of EYFP in *C. fritschii* PCC 9212, far-red light illumination caused a 30-fold increase in EYFP fluorescence, although this level was not sustained and dropped steadily over several days. EYFP fluorescence was then significantly reduced by switching the light source back to white light, with a 60%–70% decrease within 12 h. It remains to be investigated whether the deactivation was caused by a specific light wavelength within the white light or simply by the absence of far-red light.

The characterisation of de-activation kinetics of CcaS/CcaR system in this study adds new information on the reversibility of target expression which will be important for flexible metabolic engineering. When measuring the kinetics of the decrease in CcaS/CcaR system activity under red light, the relatively slow deactivation suggested that the stability of the GFP RNA transcript was a rate-limiting factor (Figure 7). Thus, the transcriptional output of the system will present different dynamics depending on the target gene expressed. Several cellular mechanisms can affect the mRNA transcript stability, one of which is the cleavage of AU-rich regions by cyanobacterial RNase E (Horie et al., 2007). The lower the frequency of AU-rich regions encoded within a gene, the longer the potential half-life of the mRNA. Some genes also encode antisense RNAs on the opposite strand, which when transcribed can bind to these AU-rich regions of the single-stranded mRNA upstream of the RBS. This prevents RNase E cleavage whilst still allowing mRNA translation, as has been shown for *PsbA* expression in *Synechocystis* sp. PCC 6803 (Sakurai et al., 2012). Therefore, when porting optogenetic tools between different species, it is important to consider the presence of promoters which may drive antisense RNA expression.

The present work is also new insofar as we tested the effect of multiple red and green light periods on CcaS/CcaR system output in cyanobacteria (Figure 8). Such experiments have been performed in *E. coli* and the obtained information is critical for the usage of an optogenetic system in the dynamic control of metabolic pathways (Fernandez-Rodriguez et al., 2017). In this context, it is also important to understand whether similar levels of activation and deactivation of gene expression can be achieved over repetitive cycles of ON/OFF switching. In measuring GFP mRNA transcript levels over three 24-h green-red cycles, two interesting observations were made (Figure 8). Firstly, the red light treatment significantly reversed the green light activation but did not reach the baseline expression level recorded before the initial green light treatment. Secondly, the level of green light activation increased from the first to the second treatment but not any further in the third treatment. It is possible that the initial 24 h of green light have a ‘priming’ effect, allowing the system to respond stronger to subsequent green light illumination. Interestingly, the priming effect was not apparent during the light/dark cycle experiment, where the difference between the 16 and 40-hour time points was not statistically significant (Figure 9). The molecular mechanisms behind a potential priming effect are unclear. One possibility is that it is simply due to higher residual GFP transcript after the 24-hour red light period. However, if this was true, an even greater increase in GFP expression after the third period of green light would be expected as residual GFP expression was higher after the second period of red light than the first. Other possible explanations are that after the first periods of red and green light, CcaR, other general transcriptional initiation factors, or RNAP remain near pCpcG2 and/or in a pre-activated state.

Previous work in cyanobacteria characterised CcaS/CcaR system activity under constant light conditions. However, cyanobacteria are often grown under light/dark cycles which is an important consideration for the deployment of optogenetics. The present work offers new information on the effect of light/dark cycles on the CcaS/CcaR system in cyanobacteria. The reduction in GFP RNA transcript in darkness appeared to be slower than under red light (Figure 7 compared to Figure 9). In darkness, CcaS will remain active until it naturally reverts to the dark state, and the observed reduction of 25%–30% suggests that the ON state half-life if the green activated CcaS/CcaR system is more than 8 h. Thus, product synthesis will continue at least to some extent during the night period. If fast deactivation of the system is desired, red light should be used.

### 4.3 CcaS/CcaR system optimisation

Previously, work has been carried out to improve the performance of the CcaS/CcaR system in *E. coli*. Truncation of pCpcG2 from 238 bp to 172 bp resulted in a modest reduction in activity under green light and a much stronger reduction of promoter activity under red light, presumably due to the removal of a predicted weak constitutive promoter (Schmidl et al., 2014). The modification thus reduced the ‘leakiness’ of the system and improved the dynamic range. In contrast, in *Synechococcus* sp. PCC 7002 the truncation of pCpcG2 resulted in increased system activity under both red and green light (Figure 10) suggesting that the removed 66 bp region contains a sequence which influences promoter activity under both red and green light in *Synechococcus* sp. PCC 7002. This underscores the differences of transcriptional regulation of heterologous systems between different bacterial hosts which may limit the transferability of some optogenetic tools between species.

pUC-172-10-GFP, which contained a modified −10 hexamer in addition to the truncated pCpcG2, showed both the largest dynamic range and the highest activity under green light of the five constructs tested, 3-fold higher than the original system (Figure 10). Modifying the −10 hexamer to match that of the consensus sequence also resulted in a 3-fold increase in pCpcG2 activity under green light in *B. subtilis* (Castillo-Hair et al., 2019). Optogenetic tools which enable high levels of transgene expression are advantageous in metabolic engineering as expression can always be lowered by reducing light intensity or duration.

To improve the transferability of optogenetic tools between species, modular transcription factor domain swapping could be used. In *E. coli,* the DNA-binding domains of OmpR family transcription factor domains can be swapped with other OmpR family members, whilst retaining similar or improved levels of transcriptional output (Schmidl et al., 2019). Indeed, the DNA binding domain of the OmpR-like transcriptional regulator CcaR was fused to the REC domain of the transcriptional regulator TorR. This allowed transcription from pCpcG2 to be controlled by the action TMAO-sensing histidine kinase TorS, which normally exhibits kinase and phosphatase activity towards the transcription factor TorR. As the present work has shown the CcaS/CcaR system is functional in *Synechococcus* sp. PCC 7002, this strategy could be adapted to allow transcription from pCpcG2 to be controlled by photoreceptor histidine kinases which respond to light wavelengths other than green or red. This would allow the use of two-component systems for optogenetics for which either the output promoter is unknown or is non-functional in *Synechococcus* sp. PCC 7002. This could be a useful general strategy for improving the transferability of systems across species.

It is important to also highlight the potential disadvantages of applying optogenetic tools in cyanobacteria. One complicating factor is that cyanobacteria rely on light for growth. This means that the wavelengths required for photosynthesis should not overlap with the wavelengths used for optogenetic control. Removing specific wavelengths from the photosynthetic light source, usually white light, could have negative effects on culture growth. There is also the possibility of crosstalk between the optogenetic system components and native light-controlled regulatory mechanisms. Finally, optogenetic systems are not always completely portable between species, as has been demonstrated in this work.

Collectively, this work demonstrates some of the challenges faced when transferring genetic tools between species. The YF1/FixJ system performed poorly in *Synechococcus* sp. PCC 7002, which contrasts with the effective CcaS/CcaR system. The detailed characterisation of the system, as well as the production of a new variant with improved performance, are important steps towards realising the full potential of optogenetics to enhance cyanobacterial biotechnology.

## Data Availability

The original contributions presented in the study are included in the article/Supplementary Material, further inquiries can be directed to the corresponding author/s.
