# Supplementary material for: Optogenetic control of gene expression in the cyanobacterium Synechococcus sp. PCC 7002
Source: Front Bioeng Biotechnol. 2025 Jan 17;12:1529022. doi: 10.3389/fbioe.2024.1529022 (PMC11782128; doi:10.3389/fbioe.2024.1529022)
Supplement: Supplementary file 3 [file DataSheet1.docx]

Supplementary Material

## Supplementary Figures


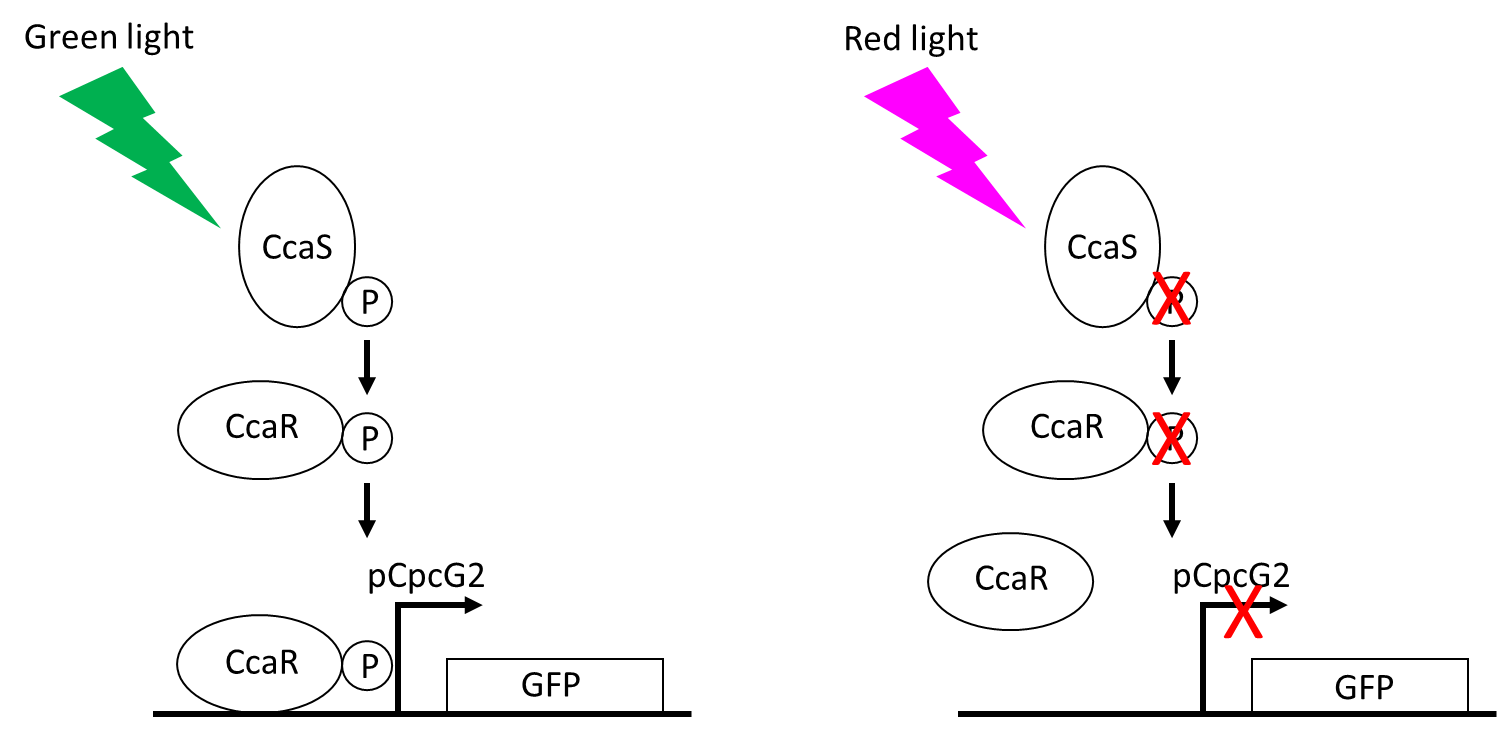
**Supplementary Figure S1. CcaS system function under green and red light.** Upon green light absorption (λ_max_ = 535 nm), the CcaS photoreceptor becomes autophosphorylated and phosphorylates the transcriptional activator CcaR. Phosphorylated CcaR binds to the G-box region of the pCpcG2 promoter, activating the transcription of the target gene. For ease of measurement, the native target CpcG2 can be replaced with the sequence for Green Fluorescent Protein (GFP) as shown here. Upon red light absorption (λ_max_ = 672 nm), CcaS becomes dephosphorylated, dephosphorylates CcaR and transcription from pCpcG2 is reduced.


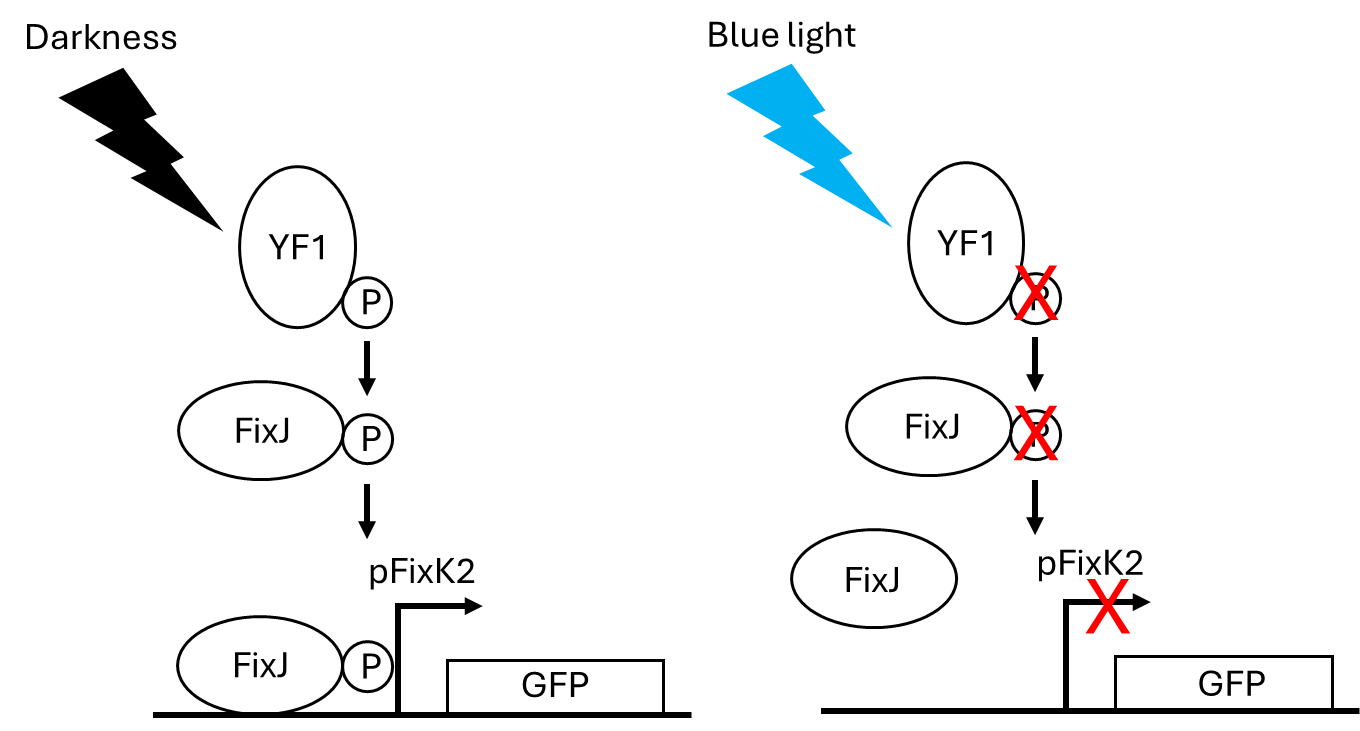


**Supplementary Figure S2. YF1 system function under blue light and darkness.** In darkness, the YF1 photoreceptor becomes autophosphorylated and phosphorylates the transcriptional activator FixJ. Phosphorylated FixJ binds to the pFixK2 promoter, activating the transcription of the target gene. For ease of measurement, the native target can be replaced with the sequence for Green Fluorescent Protein (GFP) as shown here. Upon blue light absorption (λ_max_ = 450 nm), YF1 becomes dephosphorylated, dephosphorylates FixJ and transcription from pFixK2 is reduced.


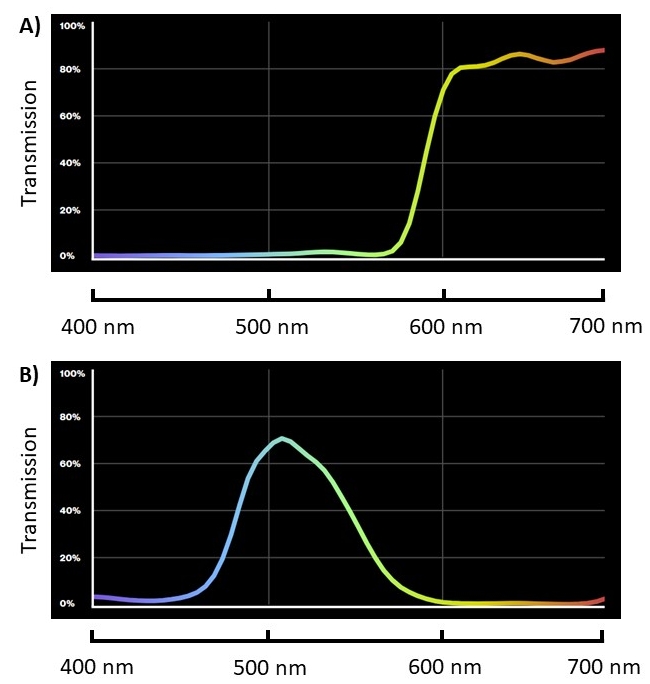


**Supplementary Figure S3. Light transmission spectra of red and green light transmitting filters.** **A)** LEE Filters Fire 019 **B)** LEE Filters Dark Green 124. Adapted from LEE Filters, 2023.


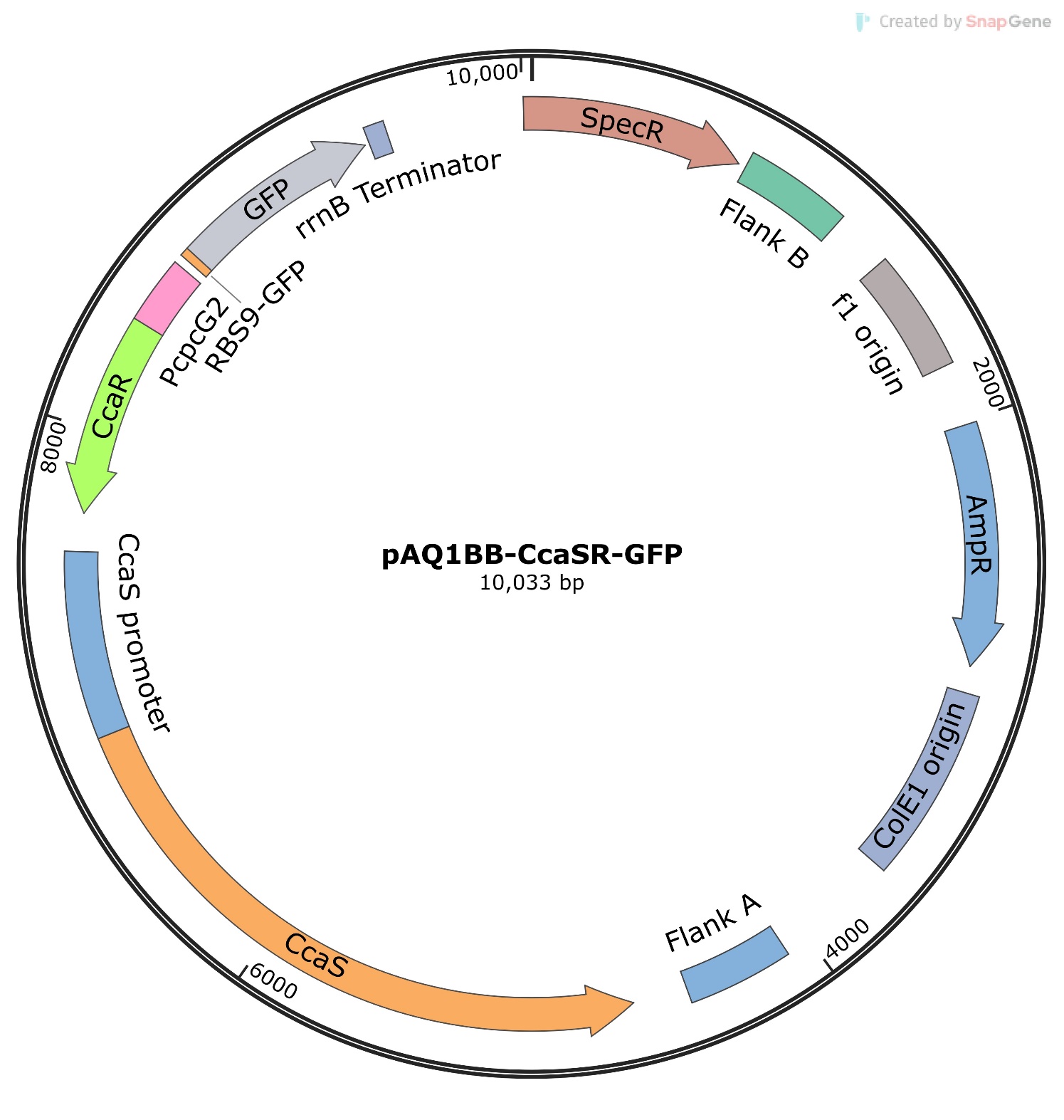


**Supplementary Figure S4**. **Plasmid map of pAQ1BB-CcaSR-GFP.** The region between Flank A and Flank B is integrated into the *Synechococcus* sp. PCC 7002 endogenous plasmid pAQ1, with the spectinomycin resistance gene SpecR allowing selection of successful transformants. The CcaR promoter overlaps with pCpcG2 on the opposite strand. The ampicillin resistance gene AmpR and the origin of replications f1 origin and colE1 origin are used for plasmid replication and selection in *E. coli*.

**Supplementary Figure S5.** **Average comprehensive gene stability ranking from RefFinder.** RefFinder Web tool output for ranking candidate qRT-PCR reference gene stability. The expression of four candidate reference genes *SYNPCC7002_A0956*, *SYNPCC7002_A1356*, *SYNPCC7002_A1784* and *SYNPCC7002_A2364* was measured in *Synechococcus* sp. PCC 7002 cultures grown under red or green LED light via qRT-PCR and the raw Cq values were inputted into the RefFinder tool. RefFinder integrates the results from Normfinder, BestKeeper, geNorm and the comparative delta CT method, with the lowest-ranking gene showing the most stable expression pattern (Xie *et al*., 2023).

**
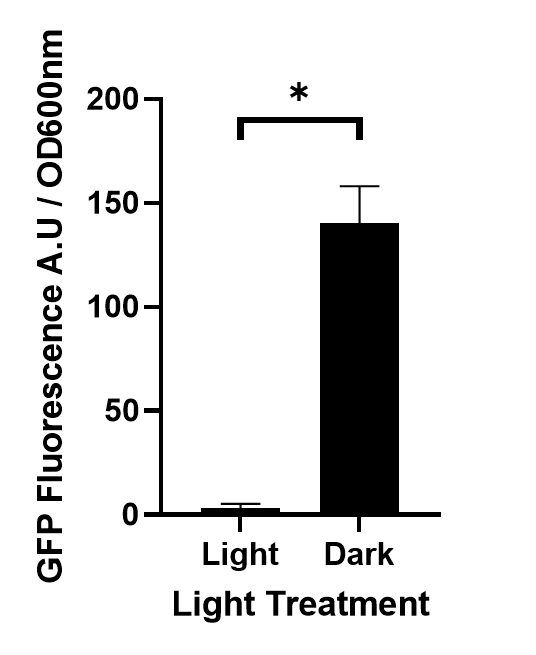
Supplementary Figure S6. YF1 system activity in *E. coli* in white light versus darkness**. 150-mL cultures of *E. coli* expressing the pDusk-GFP plasmid were grown to OD_600_ of ~0.3 under 100 µmol m^-2^ s^-1^ white LED light. The cultures were then either wrapped in foil (Dark) or left uncovered (Light). Samples were harvested from each culture 3 hours after the respective light treatments and immediately placed on ice to stop growth. GFP fluorescence was measured, normalised to OD_600_ and background fluorescence measured in cells expressing empty pDusk vector was subtracted. Data are means ± S.E.M of three independently grown cultures. Significant differences at p < 0.05 as determined by unpaired t-test are shown by asterisk.


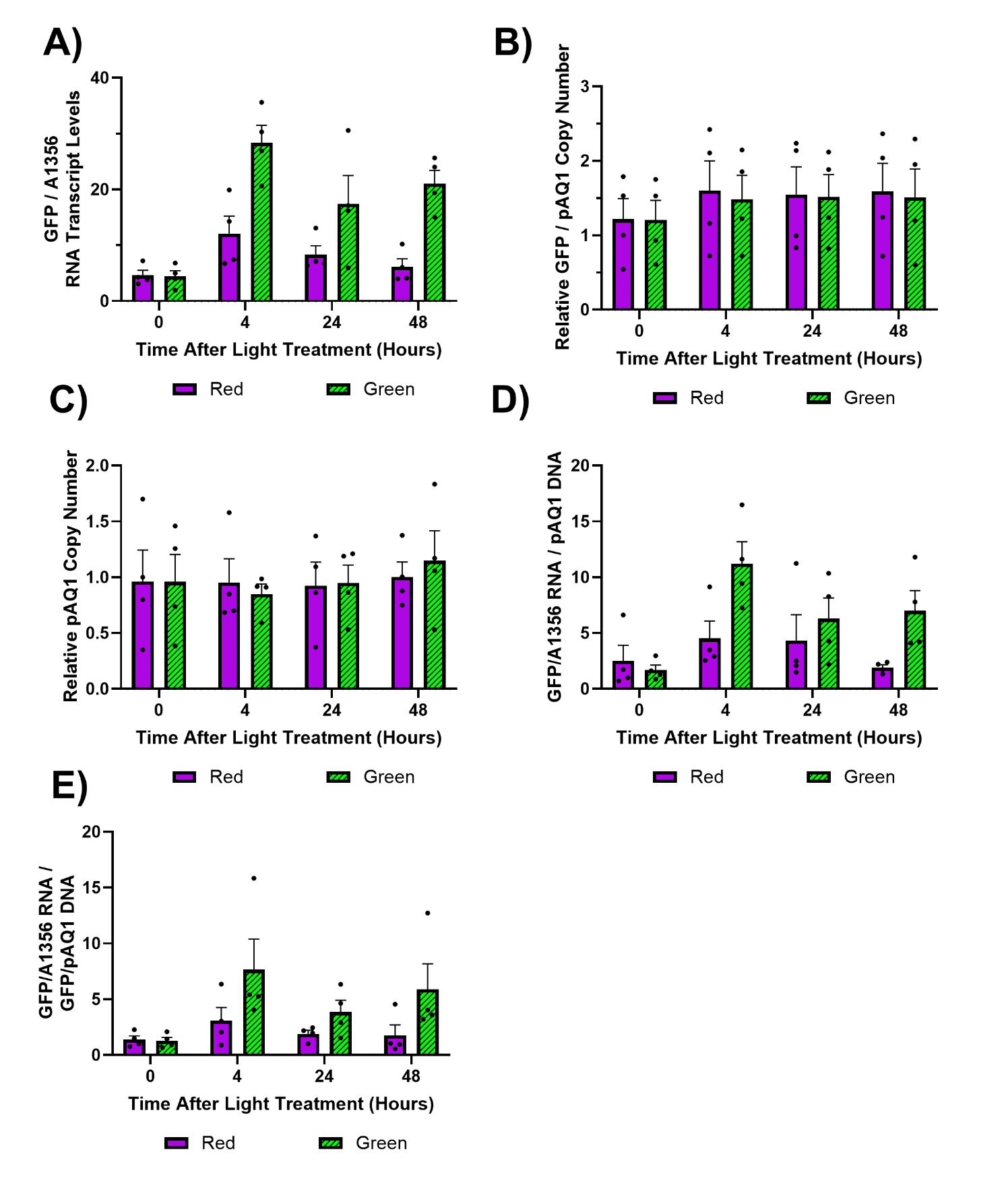


**Supplementary Figure S7. CcaS system activity as measured by qRT-PCR and normalised to DNA copy number.** 150 mL *Synechococcus* sp. PCC 7002 cultures expressing pAQ1BB-CcaSR-GFP were grown under red light for 5 days to OD_730_ = 1 under a 16/8-hour light/dark cycle. Cultures then either remained under red light or were illuminated with green light and samples as provided by combining warm white LED light with red or green light transmitting filters and were harvested at the indicated timepoints. At the 24 and 48-hour time points, samples were harvested from the cultures at the end of the 8-hour dark cycle before the lights switched back on. GFP transcript levels were measured by qRT-PCR and normalised to *SYNPCC7002_A1356*. Transgene/plasmid copy number was measured by qPCR. **A)** GFP RNA transcript levels; **B)** GFP transgene DNA copy number relative to pAQ1 copy number; **C)** pAQ1 plasmid copy number; **D)** GFP RNA transcript (normalised to *SYNPCC7002_A1356*) normalised to pAQ1 plasmid copy number; **E)** GFP RNA transcript (normalised to *SYNPCC7002_A1356*) normalised to GFP transgene DNA copy number relative to pAQ1 copy number. All data points are shown relative to the first replicate of the zero-hour timepoint in red light. Data are means ± S.E.M of four independently grown cultures. Significance at p < 0.05 was calculated using two-tailed paired t-tests.


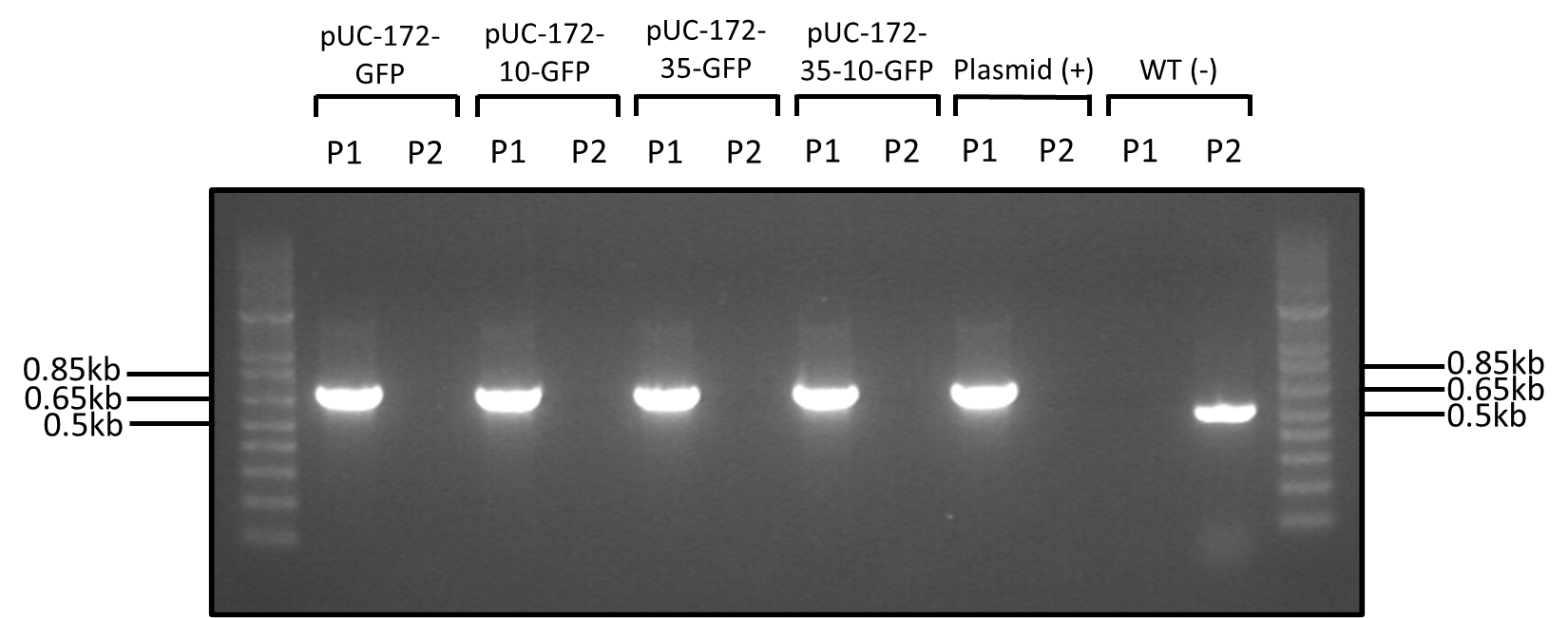


**Supplementary Figure S8. Gel image of PCR genotyping of strains containing modified pCpcG2 promoter sequences.** 300 ng gDNA extracted from *Synechococcus* sp. PCC 7002 cultures was used as a template for PCR. Reactions were run for 35 cycles and visualised on a 1.5% agarose gel stained with ethidium bromide. Primer pair 1 (P1) amplifies a short region of the Spectinomycin resistance gene, indicating successful gene integration (~700 bp). Primer pair 2 (P2) amplifies a short region of the endogenous pAQ1 plasmid which is disrupted by gene integration and thus no band should be present in a fully segregated strain. Primers flanking either side of the integrated sequence could not be used due to the large size of the insertion sequence (~7 Kb). pAQ1BB-CcaSR-GFP plasmid DNA (Plasmid (+)) was used as a positive control and wild-type *Synechococcus* sp. PCC 7002 gDNA (WT (-)) was used as a negative control.
